# Association Between the Quality of Life Domains with Anthropometric and Adiposity Indices in Rheumatoid Arthritis: A Cross-Sectional Study from a Single Centre in Erbil-Iraq

**DOI:** 10.31138/mjr.230828.jaa

**Published:** 2023-08-28

**Authors:** Aryan Mohamadfatih Jalal, Zhala Kakamin Mawlood, Sheelan Faroz Aref, Marwan Salih Al-Nimer

**Affiliations:** 1 Department of Rheumatology, Rizgary Teaching Hospital, Kurdistan Board for Medical Specialties, Erbil, Iraq,; 2 Department of Clinical Pharmacology, College of Medicine, University of Diyala, Baqubah, Iraq

**Keywords:** rheumatoid arthritis, anthropometric indices, adiposity indices, quality of life

## Abstract

**Objective:**

The aim of this study was to assess the association between the anthropometric/adiposity indices or ratios with the status of quality of life (QoL) in rheumatoid arthritis (RA) patients.

**Methods:**

This study was carried out in the Rizgary Teaching Hospital in the Kurdistan Region of Iraq between 1^st^ December 2021 and 31^st^ March 2022. Seventy-five women with a mean value of 11.3 years’ duration of disease were included in this study. The data relating to the demographic characteristics, disease activity score (DAS-28), biochemical measurements of the rheumatic profile, and anthropometric/adiposity indices and the ratios were included. The QoL of each patient was assessed using the WHOQOL-BREF.

**Results:**

The mean ± SD of the age and duration of disease were 49.6± 12.0, and 11.3±8.4 year. 70 out of 75 (93.3%) patients have a DAS-28 score of >2.6. The median values of the transform scores of the WHOQOL-BREF domains were less than 50. There were significant inverse correlations between BMI, and waist-to-height ratio with physical activity (r = −0.167, p = 0.05, and r = −0.168, p = 0.05, respectively). Social domain was significantly and inversely correlated with waist-to-hip, estimated total body fat, and waist-adjusted weight index. A higher BMI and a lower hip index were associated significantly with a lower mean scores of physical health**.**

**Conclusion:**

In RA patients, obesity is frequently observed. Over-weight and obese patients had a significantly lower mean score of physical health.

## INTRODUCTION

Rheumatoid arthritis (RA) is a systemic autoimmune disease characterised by a long duration of illness with relapse-remission phases. It commonly affects middle aged women. Obesity is a common concomitant illness in RA and the clinical manifestations of physical disability are more severe in obese RA compared with lean RA.^1^ Weight gain negatively associated with RA, as overweight-obese patients are less likely to achieve remission or get sustained remission, and they have a significant mean value of high disease activity score (DAS)-28 compared with normal weight RA.^2^

Although the circulating inflammatory biomarkers in obese RA patients are elevated, their ranges are similar to the corresponding values observed in the general population.^3,4^ A positive association of obesity on RA is the slow progression of radiological findings (synovitis) observed in obese RA patients.^5^ The relationship between obesity and the response to anti-rheumatic medicines showed conflicting results in many clinical trials.^2,6,7^ Interestingly, the anti-rheumatic medicines have an effect on the body composition as tumour necrosis factor-α (TNF) inhibitors increase visceral adipose tissue, while interleukin (IL)-6 inhibitors increase the lean muscle and inhibit the fat accumulation.^8,9^ In one systematic review and meta-analysis study, 9 out of 19 studies showed that TNF inhibitors significantly altered the body composition of RA patients, characterised by increasing their body fat mass, and two studies showed an increase in visceral adiposity with using TNF inhibitors.^10^ Interleukin-6 inhibitors that used in the management of RA can elevate the fasting serum levels of low density lipoprotein.^11^ Long-term treatment of RA patients with tocilizumab results in weight gain without increasing body fat mass and a significant gain in appendicular and skeletal muscle mass.^9^ Another cohort study extended to one year showed that tofacitinib, a Janus kinase (JAK) inhibitor, increases body weight ranging from 1 to 12 kg.^12^ Another experimental animal study showed that tofacitinib treatment for 70 days did not induce changes in body weight or compositions.^13^ Tofacitinib as add-on therapy to methotrexate significantly increases the fat mass in RA patients.^13^

Therefore, weight gain is a common finding in RA management and it is related to the suppression of inflammation.^14^ Nikiphorou et al. reported important findings that showed a link between obesity and quality of life (QoL) in 2386 newly diagnosed RA patients; they found worse QoL scores and higher DAS-28 scores after two but not five years from the diagnosis in obese patients.^15^ It has been found that obese people showed significantly lower QoL domain scores, which were not related to physical performance.^16^ Also, adiposity is a major concern in lower QoL domain scores, and the more severe the adiposity symptoms (eg, heaviness, swelling), the lower the QoL.^17^ As a result, the rationale for this study is to determine whether the status of QoL in RA patients is related to obesity/adiposity that associated with RA, as it is well known that there is an association between the disease activity with the impairment of the quality of life in RA. Therefore, this cross-sectional study was carried out in the Kurdistan region, Iraq, to assess the relationship between QoL and anthropometric and adiposity determinants in women with chronic RA.

## MATERIALS AND METHODS

From December 1^st^, 2021 to March 31^st^, 2022, a single-centre, cross-sectional study was conducted at the Rizgary Teaching Hospital (Hawler Medical University) in the Kurdistan region of Iraq. The institutional ethical committee at the Kurdistan Board for Medical Specialties in Erbil approved this work. A consent form was obtained from each patient who was willing to be included in this study. The researchers interviewed each participant and explained to them, the design and instructions of the study; they informed them that no medical interventions would be made, and that the patient was free to withdraw from the study at any time.

### Participants

Known cases of RA, who attended the consultant clinic of rheumatology in the Rizgary Teaching Hospital, were recruited for this study. The diagnosis of RA was based on the American College of Rheumatology/European League Against Rheumatism (ACR/EULAR) 2010 classification criteria.^18^ The criteria for inclusion were women with RA, aged >20 years. Patients with overt extraarticular manifestations, recent infections, cardiovascular illness, thyroid gland dysfunction, kidney and liver diseases, pregnancy, and terminal illnesses were excluded from the study.

The authors examined each patient thoroughly, and obtained their demographic characteristics. The disease activity score (DAS)-28 of 28 joints adjusted to erythrocyte sedimentation rate (ESR) was assessed and calculated by three independent practicing clinicians in rheumatology. A score of 28 joints was calculated and a cutoff value of >2.6 indicated active disease. The researchers interviewed each patient and completed the questions directed by World Health Organisation-quality of life- (WHOQOL-BREF)^19^ survey questionnaires using the native Kurdish language to explain each item of the questionnaire, including. The QoL-RA II scale consists of eight questions, and for each positive answer, a score of +1 was reported. The WHOQOL-BREF questionnaire, composed of 26 items, covers four domains; physical health, psychological, social interaction, and environmental. The calculated raw scores of 26 items were transformed to 100 scores using the manual instructions of WHOQOL-BREF. Anthropometric measurements were determined, and these included weight (kg), height (m), waist circumference (WC) (cm), and hip circumference (HC) (cm). Body mass index and lipid profile measurements were constrained in earlier studies because they were linked to RA. In this study, we measured a number of indices that accurately depicted the relationship between RA and adiposity and obesity. SThe following anthropometric and adiposity indices and ratios were calculated: waist-to- hip ratio (WHR), waist to height ratio (WHeR),^20^ conicity index,^21^ hip index (HI),^22^ waist-adjusted weight index (WWI),^23^ lipid accumulation product (LAP),^24^ visceral adiposity index (VAI),^24^ a body shape index (ABSI),^25^ abdominal volume index (AVI),^26^ and estimated total body fat (eTBF).^27^

Samples of the blood were obtained from the patients and sent to the biochemical and haematological laboratories in the Rizgary Teaching Hospital for determination of the rheumatic profile, including ESR, rheumatoid factor (RF), anti-cyclic citrullinated peptide (ACCP), and C-reactive protein (CRP), and biochemical tests, including fasting serum triglyceride (TG), and high-density lipoprotein-cholesterol (HDL-c). The reference normal range values of the rheumatic profile are:

The ESR is 1–20 mm/hour, the CRP is 3 mg/L, the ACCP is 0.2–5.0 U/mL, and the RF is 1–19 U/mL.

### Sample size

The GPower version 3.1 program, which was developed by Heinrich-Heine-Universität Düsseldorf (Germany), was applied to calculate the sample. This program is available free of charge via the Internet for both Windows and Mac OS X platforms. To show the difference between independent variables and the correlations between variables, a type I error (error probability of 0.05) and a type II error (1- error or the power) of 0.95 were adjusted. A sample size of 50 female participants is enough to detect significant changes.

### Statistical analysis

Results are presented as mean ± SD, frequencies, percentages, median, and interquartile. The relationship between the disease activity score with rheumatic profile and the domains of the WHOQOL-BREF was determined by applying the bivariant correlation test and estimating Kendall’s tau-b correlation factor and the probability. One-way Analysis of Variance (ANOVA) with *post hoc* Bonferroni test was used to compare the mean ± SD of each domain of the WHOQOL-BREF transformed score of patients with the cutoff median values of some anthropometric/adiposity indices. The median was used as a cutoff value because the reference normal values can’t be used because there are variations in these values according to age, gender, race, and ethnicity in the reported studies. A p-value of ≤ 0.05 is significant. All statistical analysis was performed, using the Statistical Packages for the Social Sciences (SPSS) version 20 (IBM-compatible Corporation; Chicago, USA).

## RESULTS

A total of 75 RA women with a mean age of 49.6 years were included in the study. The majority of patients were residents of urban areas; non-smokers; and 40% had a familial history of RA (**Table 1**). Most of the patients were presented with active RA (93.3%) by the evidence of having DAS-28 >2.6 and the rheumatic profile values, which showed a wide variation among the participants (**Table 2**). The anthropometric and adiposity indices pointed out that the patients were overweight-obese which accounted 88% of participants, and the WHeR ratio exceeded 0.6, which indicated the patients were at risk of developing cardiovascular disease (**Table 3**). **Table 4** shows that the QoL of the patients was impaired as there were 6 out of 8 domains of the QoL-RA II scale were ≤5. The mean ± SD of the WHOQOL transformed scores was less than 50%. The highest mean score was reported in the social interaction domain, and the lowest score was related to the psychological domain. **Table 5** shows that DAS-28 scores are significantly correlated with impairments of physical (r= −0.340, p<0.001), psychological (r=−0.331, p<0.001), and environmental health (r=−0.239, p=0.004). The rheumatic profile levels did not show significant correlations with QoL domains except for the ACCP levels, which were correlated positively with physical health scores (r= 0.159, p=0.025). There were significant inverse correlations between BMI and WHeR with physical activity (r= −0.167, p = 0.05 and r = −0.168, p = 0.05, respectively). Social domain significantly and inversely correlated with waist-to-hip ratio (r = −0.200, p = 0.017), estimated total body fat (r = −0.197, p=0.018), and waist-adjusted weight index (r = −0.193, p = 0.021). There were significant positive correlations between the DAS-28 score and WC (r = 0.163, p = 0.041), WHeR (r = 0.177, p = 0.026), and AVI (r = 0.161, p = 0.041).

**Table 1.** Characteristics of the participants.

**Characteristic**	**Results (n=75)**

Age (year)	49.6±12.0

Residency	
Rural	18 (24.0)
Urban	57 (76.0)

Smoking	
No	60 (80.0)
Ex-smoker	8 (10.7)
Current	7 (9.3)

Family history	
Negative	45 (60.0)
Positive	30 (40.0)

Duration of disease (years)	11.3±8.4
1–5	25 (33.3)
6–10	19 (25.3)
11–15	12 (16.0)
>15	19 (25.3)

Status of the disease	
DAS-28 score	5.5±1.7
Remission (<2.6)	5 (6.7)
Active	70 (93.3)
Low (2.6- <3.2)	5 (7.1)
Moderate (3.2–≤5.1)	16 (22.9)
High >5.1	54 (77.1)

The results are expressed as number (%) and mean ±SD.

**Table 2.** Rheumatic profile.

**Rheumatic profile**	**Mean ±SD**	**Median (interquartile)**
C-reactive protein (mg/L)	1.28±2.62	0.47(0.21–1.14)
Erythrocyte sedimentation rate (mm/h)	36.0±21.0	35 (20.0–50.0)
Rheumatoid factor (IU/mL)	71.0±132.0	21 (8.0–64.0)
Anti-cyclic citrullinated protein (U/mL)	73.2±119.9	14.58 (1.87–69.8)

**Table 3.** Anthropometric and adiposity indices of 75 participants with rheumatoid arthritis.

**Indices**	**Mean ±SD**	**Median (interquartile)**	**Number (%)**

Waist (cm)	95.2±12.6	95.0 (86.0–102.0)	
≤ 88 cm			22(29.3)
≥88 cm			53(70.7)

Body mass index (kg/m^2^)	31.6±5.9	31.2(27.0–36.1)	
Normal: 18–24 kg/m^2^			9 (12)
Overweight: 25–29.99 kg/m^2^			25 (33.3)
Obese: 30.0–39.99 kg/m^2^			33 (44)
Morbid obesity ≥40 kg/m^2^			8 (10.7)

Waist-Hip ratio	0.89±0.09	0.89 (0.83–0.95)	

Waist-Height ratio	0.612±0.085	0.600 (0.542–0.667)	

Conicity index	1.25±0.13	1.23 (1.16–1.32)	

Lipid accumulation product	18.14±6.38	17.98 (13.9–22.2)	

Visceral adiposity index	149.6±81.2	131.0 (97.8–191.8)	

A body shape index	0.077±0.008	0.078 (0.072–0.082)	

Abdominal volume index	18.7±4.8	18.3 (15.4–20.9)	

Estimated total body fat	46.8±10.0	45.8 (39.4–51.0)	

Hip index	63.5±4.9	63.1 (60.6–66.4)	

Waist-adjusted weight index (cm√kg)	10.9±1.1	10.8 (10.1–11.5)	

**Table 4.** The results of the domain scores of QoL-RA scale and WHOQOL-BREF in 75 participants with rheumatoid arthritis.

**QoL/Domains**	**Mean ± SD (Median)**	

QoL-RA (domains)		
Physical ability	4.75±2.13 (5)	
Help	6.97±2.10 (7)	
Arthritis	4.55±2.16 (4)	
Tension	5.12±2.09 (5)	
Health	4.75±1.82 (5)	
Arthritis (only)	4.61±2.02 (4)	
Interaction	6.39±2.11 (7)	
Mood	4.92±2.17 (5)	

WHOQOL-BREF (domains)	Raw score	Transformed score
Physical Health	19.8±3.11 (20)	45.61±11.40 (44)
Psychological	16.39±2.87 (16)	43.48±11.96 (44)
Social interaction	8.77±2.11 (9)	48.17±17.76 (50)
Environment	21.51±4.57 (21)	43.92±10.08 (44)

QoL: quality of life; WHO: World Health Organisation; BREF: brief questionnaire consisting of 26 items; raw score: scoring of 26 items calculation; Transformed score: the calculated score of 26 items transformed to 100.

**Table 5.** Bivariate correlations between disease activity score (DAS)-28 and rheumatic profile with the WHOQOL-BREF domains in 75 participants with rheumatoid arthritis.

**Rheumatic disease activity markers**	**Physical**	**Psychological**	**Social interaction**	**Environmental**

Disease activity score (DAS)-28	**r= −0.340**	**r= −0.331**	r= −0.157	**r= −0.239**
	**p<0.001**	**p<0.001**	p=0.059	**p=0.004**

C-reactive protein (mg/L)	r= −0.148	r= −0.072	r= −0.124	r= −0.142
	p=0.079	p=0.393	p=0.138	p=0.087

Erythrocyte sedimentation rate (mm/h)	r= −0.138	r= 0.035	r= −0.059	r= −0.054
	p=0.105	p=0.682	p=0.487	p=0.523

Rheumatoid factor (IU/mL)	r= −0.009	r= −0.058	r= −0.022	r= 0.009
	p=0.918	p=0.494	p=0.795	p=0.915

Anti−cyclic citrullinated protein (U/mL)	**r= 0.189**	r= 0.086	r= 0.060	r= 0.107
	**p=0.025**	p=0.305	p=0.473	p=0.179

r: Kendall’s tau-b correlation factors; p: probability.

Taking the cutoff value of the median of each anthropometric/adiposity indices, patients with higher values of BMI have significantly lower scores for physical health, while lower values of hip index have significantly (p=0.041) lower mean ±SD of physical health (**Table 6**).

**Table 6.** Comparisons between the domains of the quality of life taking a cutoff value of anthropometric and adiposity indices at a median level.

**Indices**	**Physical**	**Psychological**	**Social**	**Environment**

Waist circumference				
<95 cm (n=37)	48.0±11.9	42.8±12.0	50.0±16.4	43.3±13.2
≥95 cm (n=38)	42.3±10.5	44.1±12.1	46.4±19.0	44.5±15.0
	*p*=0.073	*p*=0.636	*p*=0.376	*p*=0.708

Body mass index (kg/m^2^)				
<31.2 (n=37)	48.7±11.2	44.3±12.1	49.2±18.0	45.3±14.2
≥31.2(n=38)	42.6±10.9	42.7±11.9	47.2±17.7	42.6±14.0
	***p=*0.018**	*p*=0.576	*p*=0.619	*p=*0.398

Conicity index				
<1.23 (n=36)	45.8±11.8	42.8±12.1	51.4±16.4	41.5±12.0
≥1.23(n=39)	45.4±11.1	44.1±12.0	45.2±18.6	46.1±15.6
	*p*=0.890	*p*=0.656	*p*=0.133	*p*=0.159

Lipid accumulation product				
<17.98 (n=38)	47.4±11.8	43.3±11.9	48.7±17.3	42.3±12.7
≥17.98(n=37)	43.8±10.9	43.6±12.2	47.6±18.4	45.6±15.4
	*p*=0.172	*p*=0.920	*p*=0.793	*p*=0.321

Visceral adiposity index				
<131 (n=37)	47.6±12.8	43.6±11.5	48.0±17.8	44.8±14.3
≥131 (n=38)	43.6±9.6	43.3±12.5	48.4±18.0	43.0±13.5
	*p*=0.128	*p*=0.920	*p*=0.924	*p*=0.581

A body shape index				
<0.078 (n=50)	44.8±11.3	43.5±11.3	46.2±17.7	43.8±14.4
≥0.078 (n=25)	47.2±11.7	43.4±13.4	52.1±17.5	44.1±13.7
	*p*=0.398	*p*=0.951	*p*=0.180	*p*=0.931

Abdominal volume index				
<18.3 (n=37)	45.0±10.2	42.8±11.9	46.8±18.7	43.6±14.2
≥18.3 (n=38)	46.2±12.6	44.1±12.1	49.5±16.9	44.2±14.1
	*p*=0.649	*p*=0.636	*p*=0.516	*p*=0.870

Estimated total body fat				
<45.8 (n=37)	45.1±10.4	42.4±11.7	45.4±16.8	43.3±14.8
≥45.8 (n=38)	46.1±12.4	44.5±12.3	50.8±15.3	44.6±13.5
	*p*=0.723	*p*=0.458	*p*=0.189	*p*=0.696

Hip index				
<63.1 (n=38)	43.0±11.7	41.5±11.4	48.3±19.1	41.1±13.8
≥63.1(n=37)	48.3±10.5	45.5±12.4	48.0±16.5	46.8±13.9
	*p*=0.041	*p*=0.153	*p*=0.934	*p*=0.082

Waist-adjusted weight index				
<10.8 (n=36)	45.3±10.5	42.4±11.9	45.8±20.0	43.6±14.8
≥10.8(n=39)	45.9±12.3	44.5±12.1	50.3±15.4	44.2±13.5
	*p*=0.840	*p*=0.451	*p*=0.270	*p*=0.857

Results are expressed as mean ± SD. P-value was calculated using one-way ANOVA with *post hoc* Bonferroni test.

## DISCUSSION

The results of this study showed that RA patients had low scores in the QoL domains, and the anthropometric/adiposity indices served as independent risk factors for the QoL impairment. The impairment of the QoL domains is attributed to the long-standing disease as the mean ± SD of the disease duration was 11.3 years, and to the severity of the disease activity as most of the patients had scores of DAS-28 >2.6.^28,29^ The results of this study confirmed a previous study, which showed that physical disability runs in parallel with disease duration.^28^ A significantly inverse correlation between the DAS-28 and physical domain score (r= −0.340, p<0.001) was observed in this study, which confirmed another study reported that the more severe disease activity, the higher the rate of physical disability and early retirement.^29^ The values of the rheumatic profile indicated that the patients were presented with variable degrees of disease severity, and none of them showed significant correlations with QoL domains except the levels of ACCP, which showed positive correlations with physical domain scores. The explanation for this observation is that ACCP is a diagnostic rather than a prognostic biomarker of RA.^30^ Also, this discrepancy in the ACCP levels is also reported by others who found significant high ACCP levels in RA with a lean BMI.^31^ The overall mean score of QoL among 166 Korean patients with RA, was 5.8, which is slightly higher than our results.^32^

The patients included in this study were overweight and obese because their anthropometric and adiposity indices were significantly higher than the reference values of healthy subjects. This observation confirmed other studies that showed that RA patients were overweight or obese due to several factors, including the pathogenesis of the disease and/or pharmacological interventions.^33,34^ Earlier research did not take into account changes in the body composition and lipid profile in specific indices like the VAI, LAP, and eTBF, which are more accurate than mentioning lipid profile levels or BMI. Our study demonstrates that, as shown in **Table 6**, the other indices which are related to obesity and adiposity have a non-significant association with QoL except the hip index. Obesity among RA patients is of clinical importance because it has been associated with a higher rate of arthritis activity and the patients expressed a lower response rate to TNF inhibitors.^35^

A BMI of less than 31.2 kg/m^2^ is a significant associated risk factor of physical health impairment compared to those who have greater than 31.2 kg/m^2^, indicating that obese RA patients had significantly more physical disability than overweight patients.^34,36^

A recent study demonstrated that subjects with morbid obesity (BMI≥40 kg/m^2^) showed the physical and social functioning scores, which were assessed by the Short Form-36 (SF-36) questionnaire, were higher than 50, which pointed out that the low scores of WHOQOLBREF reported in this study are related to RA.^16^

The hip index, as a determinant of hip circumference, weight, and height of the patient, showed a significant relationship with the QoL in RA. Patients with HI ≥63.1 showed higher scores in the physical health domain compared with those who had scores <63.1. Current research does not highlight the importance of using HI as an associated risk factor for RA severity. A high HI value as a marker of adiposity was not found to be an independent risk factor for developing diabetes mellitus.^37^ eTBF values at ≥45.8 were non-significantly associated with impairment of any domain of QoL. This observation confirmed other studies that showed body fat composition may be consider as an independent risk factor for RA, and that the levels of adipocytokines are significantly elevated in RA.^38,39^ Table 6 shows that the WWI (by which the waist circumference was adjusted according on the patient’s weight) was not statistically linked with QoL impairment. The interpretation of this data is that WC is not a major factor related with QoL. From table 6, we can observe that BMI and HI are significantly related factors to the impairment of the QoL compared with other indices. The study’s strength is that it emphasises that obesity and overweight are only mildly associated conditions that can impair quality of life, and that there is no specific anthropometric or adiposity biomarker that can be used as a risk factor for the poor functioning of RA patients. The key takeaway from this study is that, in addition to disease activity, obesity/adiposity should be taken into account as an associating factor to the QoL impairment in RA patients. This can be done by measuring several related indices, such as WC, BMI, LAP, VAI, etc. One of the study’s limitations was that males were not investigated because RA affects men less frequently and it is difficult to collect a large number of participants. Another limitation of this study is that the patients were treated with different and variable antirheumatic agents and these medicines may alter the body composition and the QoL.

## CONCLUSIONS

The study’s findings demonstrate that most RA patients are overweight or obese, which has been associated with low scores in quality of life domains. Body mass index is a significant factor that associated with impairment of the physical health in rheumatoid arthritis patients.

## ETHICAL APPROVAL

This study was in accordance with the standards of the Scientific and Ethics Committees at the Kurdistan Board for Medical Specialties in Erbil, Iraq, and in accordance with the 1964 Helsinki Declaration, and informed consent was obtained from all patients included in the study.
